# Successful Treatment of Adalimumab-Induced Pustular Psoriasis With Guselkumab in a Patient With Hidradenitis Suppurativa

**DOI:** 10.7759/cureus.84626

**Published:** 2025-05-22

**Authors:** Kostandin Valle, Divya Pothuri, Travis Jackson, Ashley M Jenkins

**Affiliations:** 1 Dermatology, University of Missouri School of Medicine, Columbia, USA

**Keywords:** adalimumab (humira), biologic treatment, generalized pustular psoriasis, guselkumab, hidradenitis suppurativa, paradoxical psoriasis

## Abstract

Pustular psoriasis is a rare and severe form of psoriasis, characterized by the presence of desquamative plaques with pustules on an erythematous base. Psoriasis is thought to result from plasmacytoid dendritic cell (PDC)-mediated T-cell activation, which stimulates keratinocyte proliferation via type 1 interferon signaling. Studies suggest that TNF-alpha inhibition can paradoxically enhance interferon-alpha activity, leading to the development of pustular psoriasis in some cases. A 58-year-old patient with hidradenitis suppurativa began adalimumab therapy. One month later, she presented with a diffuse pustular rash. A punch biopsy revealed pustular psoriasis with negative direct immunofluorescence (DIF) and periodic acid-Schiff (PAS) stain. Despite treatment with topical steroids, the rash worsened. Her therapy was switched to guselkumab, alongside continued topical steroids. This resulted in significant improvement within a week, with continued resolution at the one-month follow-up. Psoriasis and hidradenitis suppurativa are driven by chronic inflammation involving TNF-alpha and the IL-23/IL-17 axis. While TNF-alpha inhibitors like adalimumab reduce inflammation, paradoxical reactions like pustular psoriasis can occur due to enhanced interferon-alpha activity. In patients on this therapy who develop a new-onset diffuse pustular rash, an index of suspicion for this condition should be maintained. TNF-alpha inhibitors should be discontinued if pustular psoriasis develops, with IL-23 inhibitors providing a viable alternative.

## Introduction

Psoriasis is a cutaneous inflammatory disease hypothesized to occur due to plasmacytoid dendritic cell (PDC)-induced T-cell mediated proliferation via stimulation by type 1 interferons. These interferons stimulate T cells, especially Th1 and Th17, to secrete pro-inflammatory cytokines such as TNF-alpha, IL-17, and IL-22. These pro-inflammatory cytokines induce the hyperproliferation of keratinocytes, leading to the characteristic thickening of the epidermis seen with the disease [1]. Pustular psoriasis is a subtype described clinically as desquamative plaques with pustules on an erythematous background. This pustular form of the disease is hypothesized to result from the relationship between the PDC-produced interferons and TNF-alpha and the upregulation of IL-36 activity, which interacts with the pro-inflammatory cytokines mentioned previously [2,3]. 

TNF-alpha in pustular psoriasis works by promoting an overall inflammatory response and recruiting neutrophils to induce the formation of pustules, which further contributes to disease pathology. TNF-alpha inhibitors thus work by blocking the action of TNF-alpha and preventing it from interacting with other cellular pathways, including the NF-kB pathway [4]. NF-kB signaling is an important inflammatory pathway that regulates the expression of genes involved in immune response and has been frequently associated with the pathogenesis of psoriasis. As such, TNF-alpha antagonists decrease inflammatory signaling cascades and the production of cytokines that would otherwise result in keratinocyte proliferation, which is abnormally increased in psoriasis. While highly effective in treating psoriasis, TNF-alpha inhibitors may not always resolve the type 1 interferon-driven component of psoriasis. 

The cross-regulation of TNF-alpha and interferon-alpha is well-characterized in the literature. The antagonism of TNF-alpha sustains the production of interferon-alpha by PDCs, which may contribute to the paradoxical reactions of TNF-alpha inhibitors [5]. One study showed that skin lesions in psoriasis patients treated with TNF-alpha inhibitors had increased levels of PDCs and type 1 interferons [6]. Additionally, TNF-alpha inhibitors have been shown to induce and exacerbate psoriatic skin lesions in up to 5.3% of patients [7]. Adalimumab is a humanized mouse IgG1 monoclonal antibody that binds to TNF-alpha and inhibits its effects on TNFR1 and TNFR2 receptors [8]. In addition to treating psoriasis, TNF-alpha inhibitors are often used as first-line biologic treatments for patients with hidradenitis suppurativa. 

Hidradenitis suppurativa and psoriasis share common inflammatory pathways, including the TNF-alpha and IL-23/IL-17 axis. Additionally, the pathogenesis of pustular psoriasis involves the upregulation of IL-36, which can be induced by IL-17 signaling and worsen the disease process [9]. While TNF-alpha inhibitors like adalimumab target upstream inflammatory mediators, IL-23 inhibitors such as guselkumab offer a more selective approach. IL-23 inhibitors effectively reduce keratinocyte proliferation and inflammation associated with hidradenitis suppurativa and psoriasis by inhibiting the IL-23/IL-17 axis, therefore affecting the neutrophil recruitment and IL-36 signaling that leads to the pustular eruption [9]. Thus, IL-23 inhibitors may offer multimodal approaches to therapy for patients with hidradenitis suppurativa and pustular psoriasis by targeting cytokines involved in these various inflammatory pathways. We present a case highlighting the efficacy of guselkumab in addressing the inflammatory overlap between these two conditions, providing resolution of both hidradenitis suppurativa and paradoxical pustular psoriasis. 

## Case presentation

**Figure 1:**
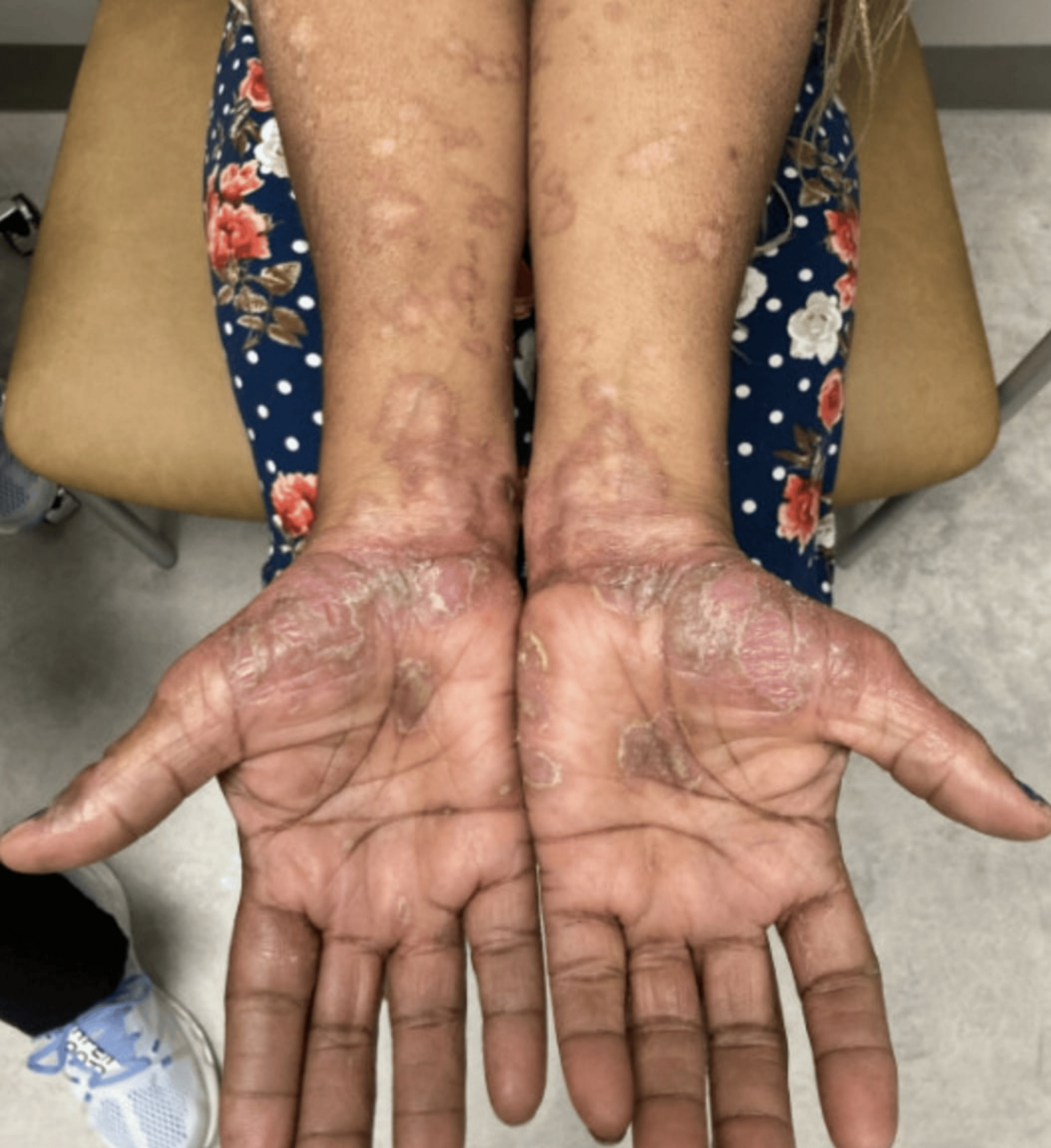
Diffuse desquamative pustular rash with underlying annular erythematous plaques on the bilateral forearms.

**Figure 2:**
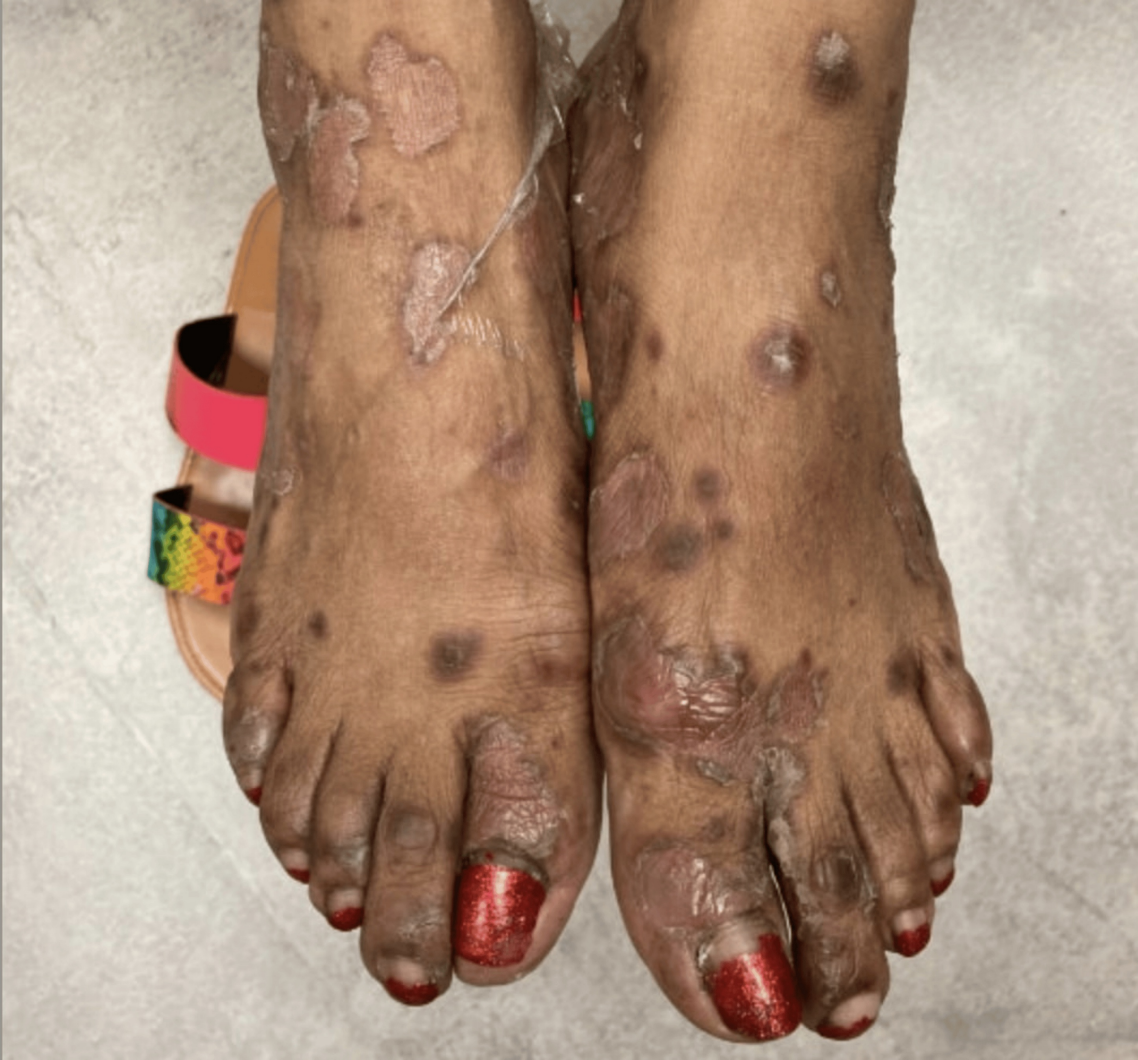
Diffuse desquamative pustular rash with underlying erythematous plaques on the bilateral feet.

A 58-year-old Black woman with a history of hidradenitis suppurativa presented to our clinic for worsening symptoms. The patient had tried the standard treatment algorithm and thus was started on adalimumab therapy. An initial loading dosage of 160 mg subcutaneous adalimumab followed by 80 mg subcutaneous injections every other week was attempted. However, she continued to experience symptoms, and at her follow-up appointment three months later, her dose was amended to 40 mg of subcutaneous adalimumab injections weekly. One month later, she returned to our clinic with new-onset "pus-filled bumps" that initially appeared on her axillae shortly after the treatment regimen was changed, but had since spread diffusely throughout her body after the treatment regimen was changed, with significant prominence on the wrists and feet (Figures 1-2). The patient denied any prior history of psoriasis. A physical exam revealed numerous diffusely scattered minute non-follicular sterile pustules with an annular configuration overlying erythematous plaques on her chest, back, palms, and insteps of her soles. Vital signs were within normal limits, and the patient was afebrile. 

A punch biopsy was performed on the patient's right wrist, which revealed eruptive pustular psoriasis with negative direct immunofluorescence (DIF). Additionally, a periodic acid-Schiff (PAS) stain was negative for dermatophytes. Acute generalized exanthematous pustulosis and subcorneal pustular dermatosis were considered as a differential but were not favored given the psoriasiform epidermal hyperplasia seen concurrently. The patient was started on clobetasol 0.05% topical ointment and 0.1% triamcinolone cream, with discontinuation of adalimumab. 

**Figure 3:**
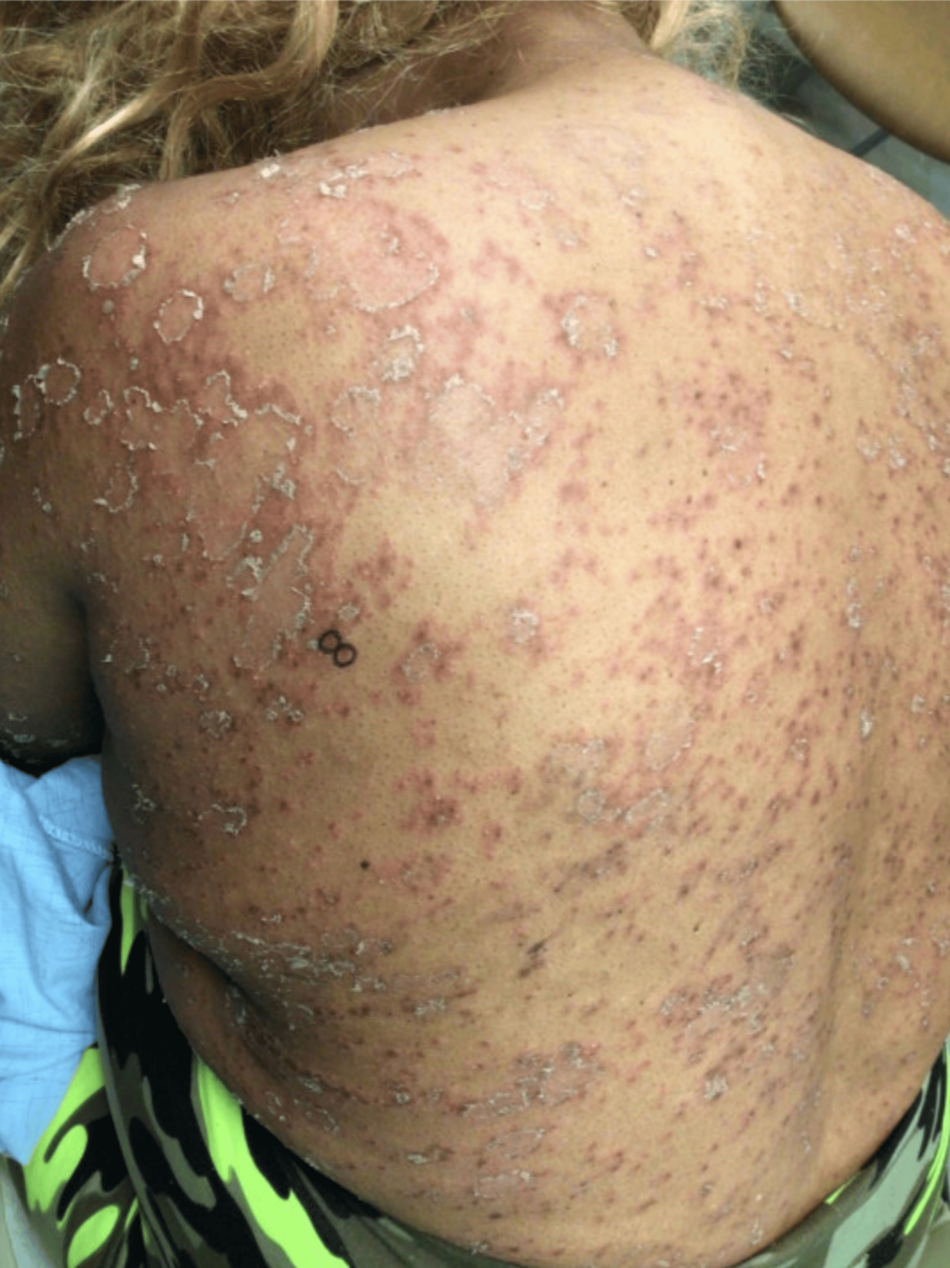
Desquamative pustular eruption scattered on the back with underlying erythematous plaques.

Two weeks later, the patient returned to the clinic with a continually worsening rash and no improvement using topical steroids. A repeat punch biopsy was performed on the patient's left lateral upper back (Figure 3). Hematoxylin and eosin (H&E) staining showed neutrophilic debris and focal parakeratosis in the stratum corneum with slight diffuse spongiosis. PAS and DIF stains were negative once again. These findings, along with the clinical picture, were consistent with the diagnosis of pustular psoriasis. The treatment regimen at this time was amended to guselkumab 100 mg subcutaneous injection in the clinic, triamcinolone 0.1% cream twice a day, and a follow-up appointment in one week. 

**Figure 4:**
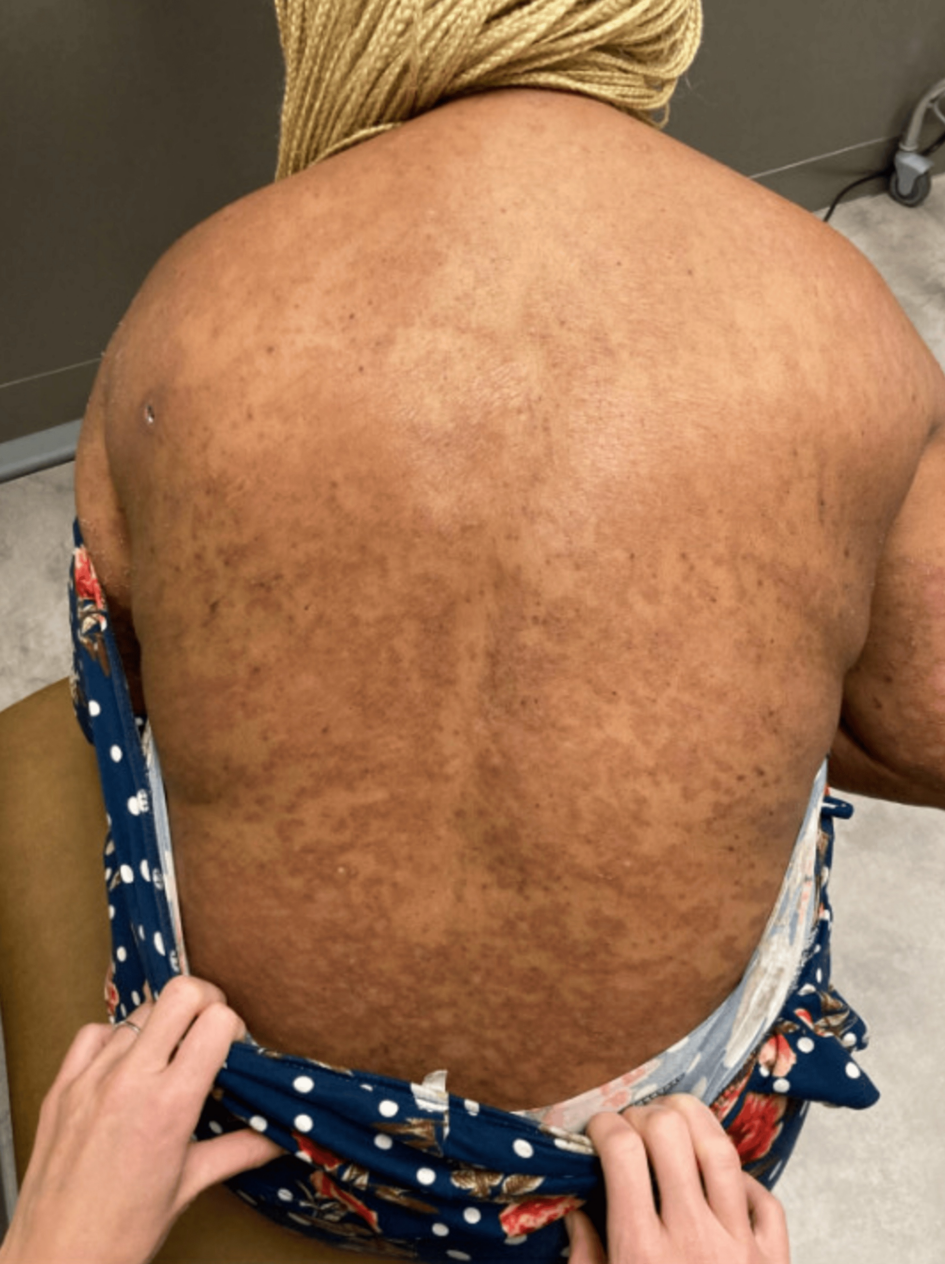
Clinical improvement following guselkumab treatment with persisting post-inflammatory hyperpigmentation.

Upon returning to the clinic, the patient noted great improvement in her clinical condition (Figure 4). She did not note any adverse reactions or side effects from treatment. Physical examination revealed thin, scattered, erythematous plaques in an annular configuration with overlying desquamation most prominent on the legs, hands, and feet. Hyperpigmented patches in similar configurations were noted on the patient's back and arms. No involvement of the face, upper chest, or neck was noticed on the physical exam, and no active pustules were present. Due to her improvement, the treatment regimen consisted of guselkumab 100 mg subcutaneous injection at week 4 and then every eight weeks after that. Additionally, the patient was told to continue applying clobetasol 0.05% topical ointment and 0.1% triamcinolone cream to affected areas. 

On her clinic visit one month later, the patient was happy with her improvement and continued guselkumab, endorsing the cessation of topical steroids as adjunct therapy for the control of her pustular psoriasis. Her physical exam was remarkable for erythematous annular patches with peripheral desquamative scale on the plantar feet and palmar hands. Since then, the patient has not returned to the clinic to be seen. 

## Discussion

Psoriasis and hidradenitis suppurativa are driven by chronic inflammation involving TNF-alpha and the IL-23/IL-17 axis [10,11]. While TNF-alpha inhibitors like adalimumab reduce inflammation, paradoxical reactions like pustular psoriasis are a well-documented sequela that can occur due to enhanced interferon-alpha activity [12]. In patients on this therapy who develop a new-onset diffuse pustular rash, a heightened index of suspicion for this condition should be maintained. Histopathology can support the diagnosis by revealing neutrophilic debris with focal parakeratosis in the stratum corneum and diffuse spongiosis [13]. If pustular psoriasis develops, cessation of TNF-alpha inhibitor therapy is imperative in reversing symptoms, and alternative treatments should be pursued. A promising alternative is guselkumab, a monoclonal antibody that selectively targets the p19 subunit of IL-23 [14]. IL-23 inhibitors like guselkumab have been associated with a lower risk of paradoxical cutaneous reactions compared to other biologic therapies. This suggests a more favorable safety profile with these medications when treating TNF-alpha inhibitor-induced reactions [15]. In addition to this beneficial safety profile, guselkumab has also been shown to improve the management of patients with hidradenitis suppurativa who have not responded to other biologic treatments. In a recent multicenter retrospective study involving 69 individuals with severe hidradenitis suppurativa, there were no reported serious adverse events over a 48-week treatment period with guselkumab [16]. Although more studies evaluating long-term safety data are needed, IL-23 inhibitors such as guselkumab have been shown in multiple studies to be effective and have favorable safety profiles.

## Conclusions

Paradoxical pustular psoriasis induced by adalimumab presents a significant hurdle for patients receiving therapy for hidradenitis suppurativa. This condition results from complex interactions between various inflammatory pathways, cytokines, and interleukins. Both conditions share common inflammatory pathways, including the TNF-alpha and IL-23/IL-17 axis. Additionally, pustular psoriasis pathogenesis involves the upregulation of IL-36 signaling. Although inhibition of TNF-alpha is beneficial in hidradenitis suppurativa patients, the blockade of this inflammatory pathway can lead to the dysregulation of other cytokines and the subsequent paradoxical reaction seen in this patient. Prompt cessation of adalimumab and replacement with another medication are essential to treat the pustular psoriasis and to prevent the worsening of hidradenitis suppurativa. Guselkumab is a viable alternative option for these patients. By inhibiting IL-23, guselkumab downregulates Th17 activation, reducing keratinocyte hyperproliferation and inflammation in both psoriasis and hidradenitis suppurativa. Guselkumab's inhibition of this axis may also play a role in reducing signaling of IL-36, which is involved in the pathogenesis of pustular psoriasis. Thus, targeted therapy with guselkumab can offer effective management of both conditions without triggering interferon-driven inflammatory reactions.
